# Lipid, Aqueous and Mucin Tear Film Layer Stability and Permanence within 0.15% Liposome Crosslinked Hyaluronic Acid versus 0.15% Non-Crosslinked Hyaluronic Acid Measured with a Novel Non-Invasive Ocular Surface Analyzer

**DOI:** 10.3390/jcm11133719

**Published:** 2022-06-27

**Authors:** José-María Sánchez-González, Concepción De-Hita-Cantalejo, Concepción Martínez-Lara, María Carmen Sánchez-González

**Affiliations:** 1 Department of Physics of Condensed Matter, Optics Area, Vision Sciences Research Group (CIVIUS), Pharmacy School, University of Seville, 41012 Seville, Spain; mhita@us.es (C.D.-H.-C.); msanchez77@us.es (M.C.S.-G.); 2 Department of Nursing, University Hospital Virgen Macarena, University of Seville, 41009 Seville, Spain; cmartinez6@us.es; 3 Nursing Department, Faculty of Nursing, Physiotherapy and Podiatry, University of Seville, 41009 Seville, Spain

**Keywords:** tear film stability, tear film permanence, hyaluronic acid, crosslinked hyaluronic acid, liposome eyedrops, dry eye disease

## Abstract

To evaluate the stability and permanence of the liquid film created after the instillation of 0.15% crosslinked hyaluronic acid with liposomes and crocin versus the effect of 0.15% standard hyaluronic acid, a prospective, longitudinal, single-blind, single-center study was conducted in symptomatic populations with a novel noninvasive ocular surface analyzer. Limbal and bulbar redness classification, lipid layer thickness, tear meniscus height, and first and mean noninvasive break-up time (FNIBUT and MNIBUT) were performed before and 30 and 45 min after liposome-crosslinked hyaluronic acid (LCHA) and standard hyaluronic acid (HA) eye drop instillations. LCHA had a higher lipid layer thickness than HA (grades 2.00 ± 0.83 and 1.17 ± 0.63 on the Guillon pattern, respectively). LCHA achieved a better tear meniscus height than HA (0.23 ± 0.02 and 0.21 ± 0.02 mm, respectively). LCHA improved FNIBUT and MNIBUT more than HA (for FNIBUT, 6.30 ± 0.94 and 4.77 ± 0.89 s, respectively. For MNIBUT, 17.23 ± 5.11 and 12.41 ± 4.18 s, respectively). Crosslinking hyaluronic acid with liposomes and crocin significantly increases the permanence and stability of the lipid, aqueous, and mucin tear film layers. In a short-term period, liposome and crosslinked hyaluronic acid achieved better first and mean noninvasive break-up times than standard hyaluronic acid.

## 1. Introduction

Dry eye disease (DED) is usually a consequence of several related factors. On the one hand, abnormal physiology of the Meibomian glands and reduced tear production or excessive evaporation produce evaporative DED and aqueous-deficient DED, respectively. The heterogeneous nature of disease and the variability of signs and symptoms do not allow an accurate diagnosis. Earlier studies have measured tear meniscus height using the Schirmer test and tear break-up time (BUT) invasively using fluorescein [1,2,3].

The use of non-invasive diagnostic tests that improve the repeatability of measurement and diagnosis is recommended, and comprehensive DED treatment is jointly considering the symptoms and all possible etiologies [4]. Included among the non-invasive instruments for dry eye measurement are the LipiView^®^ interferometer (TearScience Inc., Morrisville, NC, USA), the IDRA^®^ ocular surface analyzer from SBM System^®^ (Orbassano, Torino, Italy), and the Ocular Surface Analyser (OSA) from SBM System^®^ (Orbassano, Torino, Italy) [5,6,7]. The OSA is an instrument very similar to IDRA; however, IDRA performed an automatic tear meniscus measurement, while OSA does it manually. Lipi View II is a limited instrument that does not measure subjective questionnaire, conjunctival hyperemia, or NIBUT. In our study, an automated, non-invasive diagnosis of the ocular surface was performed with the OSA. The OSA allows to identify the type of DED and which layers of the tear should be treated according to the type of deficiency [5,8].

Artificial tears are used in the treatment of dry eye as a substitute for the tear film. Usually, they all have an aqueous base to which different molecules are added to improve their lubrication, viscosity, osmolarity, tolerance, and residence time on the ocular surface [9]. Many of the tear preparations include viscosifying agents in their composition that function as a lubricant and increase the permanence of the ophthalmic solution on the ocular surface. There are few studies reporting the ocular surface effects of artificial tears that include Crosslinked hyaluronic acid (HA), liposomes, and crocin in their composition. That is why we carried out our study to ascertain whether there is an improvement in the anterior surface of the eye after using a tear that combines the three components.

In recent years, it is very common to include HA in the composition of artificial tears. HA is a linear polymer with a high molecular weight, a natural origin, and a high hydrophilic property. It does not generate an immune response and has a viscoelastic capacity. When formulated in eye drops, it improves the stability of the tear film by better retaining water and helps keep the ocular surface lubricated, hydrated, and protected [10,11,12,13]. In addition, it has a high regenerative, antioxidant, and anti-inflammatory capacity [14,15]. HA concentrations vary from 0.03%, 0.1%, 0.15%, and 0.18% to 0.4% [1,13] in the wide variety of commercially marketed eye drops. However, the effectiveness of hyaluronic acid decreases over time [16]. Crosslinked HA increases its molecular density and allows the effects to last longer because the body takes longer to reabsorb and degrade it, endowing it with better longevity [15].

In addition, the incorporation of antioxidant agents in the formulation of artificial tears is recommended, since oxidative stress is one of the factors involved in DED [17]. Crocin is a natural chemical compound of the carotenoid or pigment type found in the stigmas of the *Crocus sativus* or saffron flower. Specifically, it is the diester formed from the disaccharide gentiobiose and the dicarboxylic acid crocetin [18]. It belongs to a group of unusual carotenoids due to its solubility in water. In addition, its antioxidant and anti-inflammatory properties stand out [18,19], and, in aqueous solution, it is capable of increasing the viscosity and mucoadhesive properties of the preparation on the ocular surface [20].

The presence of lipids is also recommended in the formulation of tear preparations, especially when there is tear film lipid layer (TFLL) instability [2,21]. The use of liposomes, which are lipid vesicles that allow replenishment (TFLL) and reduce the surface evaporation of water, is recommended [21,22]. In addition, liposomal formulations are often used as transport vehicles for active ingredients, both hydrophilic and lipophilic, improving their bioavailability at the ocular level [21].

The combination of crosslinked HA, liposomes, and crocin should generate a synergistic action on the ocular surface, improving the stability of the tear film after instillation.

The purpose of this research was to make a non-invasive diagnosis of the ocular surface in terms of limbal and bulbar redness, lipid layer thickness, tear meniscus height, and break-up time, and to evaluate the stability and permanence of the liquid film created after the instillation of 0.15% crosslinked hyaluronic acid with liposomes and crocin versus the effect of 0.15% standard hyaluronic acid alone.

## 2. Materials and Methods

### 2.1. Design

We conducted this prospective, longitudinal, single-blind, single-center study at the Optics and Optometry Department of the Pharmacy School (University of Seville, Seville, Spain) between January and March 2022. This research was conducted according to the Helsinki Declaration and the Ethics Committee Board of the University of Seville.

### 2.2. Subjects

All of the subjects read and signed the informed consent form. An information sheet was provided to all subjects with the detailed study procedure. The inclusion criteria were as follows: (1) dry eye disease symptomatic subjects without any eye treatment, (2) age between 18 and 30 years old, (3) ocular surface disease index (OSDI) score above 5 points, (4) invasive break-up time (BUT) under 15 s, (5) subjects who completed all examination procedures, and (6) subjects who fully comprehended the purpose and methods of this research study and signed an informed consent form before the measurements. The exclusion criteria were as follows: (1) any previous eye surgery; (2) any systemic diseases; (3) any pharmacological treatment; and (4) contact lens wearers.

### 2.3. Materials

Noninvasive tear film analysis was performed with the Integrated Clinical Platform (ICP) Ocular Surface Analyzer (OSA) from SBM System^®^ (Orbassano, Torino, Italy). The OSA includes a full assessment of the ocular surface through a combination of dry eye disease diagnostic tests. The instrument is placed in the slit lamp tonometer hall. The image resolution was 6 megapixels; the acquisition mode was multishot and movie acquisition; the focus could be manual or automatic; Placido disc and NIBUT grids were available, both colored and sensitive to infrared cameras; and the light source was infrared LED or blue and white LED. Two subjective dry eye disease questionnaires were used: the Ocular Surface Disease Index (OSDI) and the Standard Patient Evaluation of Eye Dryness (SPEED) test.

Regarding the lubricants studied, eyedrop A (liposome crosslinked hyaluronic acid, LCHA group) was 0.15% crosslinked hyaluronic acid sodium salt, liposomes, crocin, ethylenediaminetetraacetic (EDTA) acid sodium salt, and 7.2 pH isotonic buffered solution with a sufficient quantity for 100 milliliters (Aquoral Lipo^®^, distributed by ESTEVE Pharmaceuticals^®^, Barcelona, Spain, and manufactured by Omisan Farmaceuti^®^, Guidonia Montecelio, Italy). This eyedrop was packaged in a multidose 10 milliliter bottle. For the control group, eyedrop B (hyaluronic acid, HA group) was 0.15% hyaluronic acid sodium salt, sodium chloride, trometamol, hydrochloric acid, and 7.2 pH isotonic buffered solution with a sufficient quantity for 100 milliliters (Hyabak^®^, Laboratories Thea, Clermont Ferrand, France). This eyedrop was packaged in a multidose 10 milliliter bottle.

### 2.4. Examination Procedure

In the first phase, subjects were included or excluded according to the previously defined criteria. The included subjects were randomized according to simple computer-generated random numbers to either eyedrop A or B. All subjects were instructed to avoid using any eye lubricants or drops one week prior to the study. After this wash-out period was finished, subjective questionnaires and a noninvasive examination with OSA were performed, from minor to major tear film fluctuations, in the following order: [1] Limbal and bulbar redness classification (LBRC) that detected the blood vessel fluidity of the conjunctiva, evaluating the redness degree with the Efron Scale (0 = normal, 1 = trace, 2 = mild, 3 = moderate and 4 = severe). [2] Lipid layer thickness (LLT) evaluation with optic interferometry, evaluating the quantity of the lipids layer into 7 different pattern categories (<15 nm—Not present, ~15 nm—Open meshwork, ~30 nm—Close meshwork, ~30/80 nm—Wave, ~80 nm—Amorphous, ~80/120 nm—Color fringes, ~120/160 nm—Abnormal color). [3] The tear meniscus height (TMH) measurement evaluates the aqueous layer quantified by a millimeter caliper (≤0.20 mm—abnormal and >0.20 mm—normal). [4] First and mean noninvasive break-up time (FNIBUT and MNIBUT) were evaluated with a special grid cone, which evaluates the quality of the mucin layer in seconds (<10 s—abnormal and ~20 s—normal).

In the second phase, the subjects were re-evaluated after 30 and 45 min to quantify the permanence and stability of the liquid film created after the instillation of both eyedrops. The temperature and humidity in the examination room were stable during all measurements.

### 2.5. Statistical Analysis

Statistical analysis was performed with SPSS statistical software (version 26.0, IBM Corp., Armonk, NY, USA). Descriptive analysis was performed with the mean ± standard deviation (SD) and (range value). The normality distribution of the data was assessed with the Shapiro–Wilk test. Differences in qualitative variables were assessed with the chi-square test. The differences between the first, second, and third OSA measurements were performed with the Wilcoxon test. Differences within both eyedrop groups were performed with the Mann–Whitney U test. The correlation study was evaluated with the Spearman Rho test. For all tests, the level of significance was established at 95% (*p* value < 0.05). The sample size was evaluated with the GRANMO^®^ calculator (Institut Municipal d’Investigació Mèdica, Barcelona, Spain. Version 7.12). A two-sided test was used. The risk of alpha and beta was set at 5% and 20%, respectively. The estimated standard deviation (SD) of the differences was set at 0.60 (based on Özgün et al. [23] SD of the main variable), the expected minimum NIBUT difference was set at 0.35 s, and, finally, the loss to follow-up rate was set at 0.10. This achieved a recommended sample size of 24 subjects.

## 3. Results

Sixty eyes from thirty patients were included in this study. Demographic and baseline data about the sex distribution, age, sphere refraction, cylinder refraction, axis refraction, CDVA (log MAR and Snellen scale), OSDI, and SPEED are presented in Table 1. None of the variables were statistically different between the 0.15% LCHA group and the 0.15% HA group. Therefore, both groups were similar and comparable at the beginning of this research.

### 3.1. Limbal and Bulbar Redness Classification

**Figure 1:**
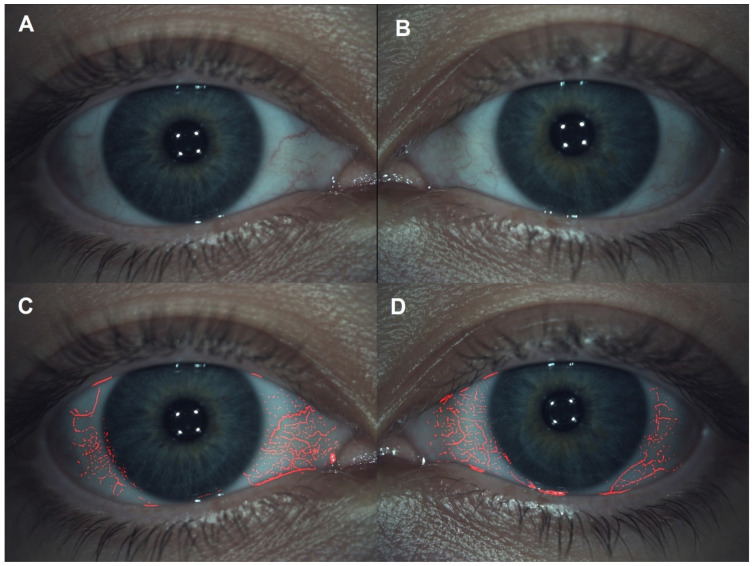
Limbal and bulbar grade 1 redness classification. (**A**,**C**) were right eyes. (**B**,**D**) were left eyes.

Blood vessel conjunctiva fluidity differences between the 0.15% LCHA group and the 0.15% standard HA group prior to eyedrop instillation and 30 min and 45 min after eyedrop instillation are presented in Table 2. In a longitudinal approach, for the 0.15% LCHA group, the change between the previous assessment and 30 min was 0.00 ± 0.58 grades on the Efron Scale (*p* = 0.99), and the change between the 30-min and 45-min assessments was 0.16 ± 0.38 grades on the Efron Scale (*p* = 0.04). Regarding the standard HA group, the change between the previous assessment and 30 min was 0.08 ± 0.28 grades on the Efron Scale (*p* = 0.15), and the change between the 30-min and 45-min assessments was 0.00 ± 0.00 grades on the Efron Scale (*p* = 0.99). Limbal and bulbar redness examination examples are presented in Figure 1.

### 3.2. Lipid Layer Thickness

**Figure 2:**
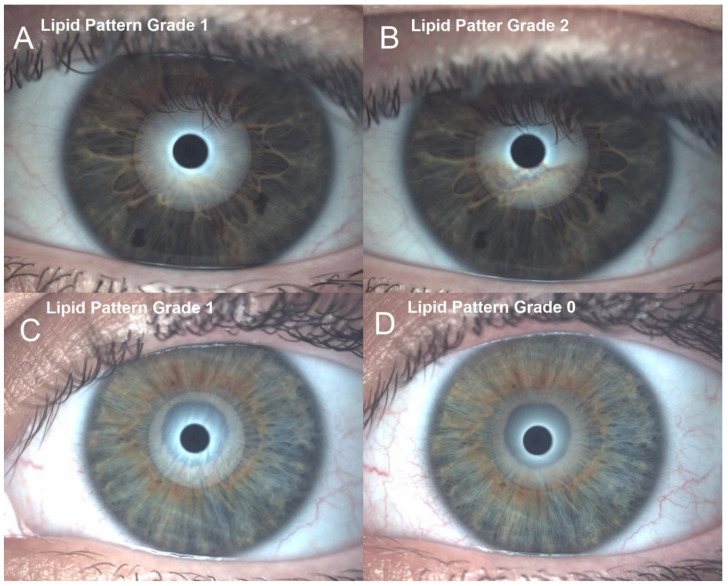
Interferometric lipid pattern differences between previous eye drop instillation (left) and 45 min after instillation (right). (**A**,**B**) CHA group before and after eyedrop. (**C**,**D**) HA group before and after eyedrop.

Interferometric lipid pattern differences between the 0.15% LCHA group and the 0.15% standard HA group prior to eyedrop instillation and 30 min and 45 min after eyedrop instillation are presented in Table 2. In a longitudinal approach, for the 0.15% LCHA group, the change between the previous assessment and 30 min was an increase of 0.83 ± 0.38 grades on the Guillon Scale (*p* < 0.01), and the change between the 30-min and 45-min assessments was 0.16 ± 0.38 grades on the Guillon Scale (*p* = 0.04). Regarding the standard HA group, the change between the previous assessment and 30 min was a decrease of 0.50 ± 0.51 grades on the Guillon Scale (*p* = 0.01), and the change between the 30-min and 45-min assessments was a decrease of 0.04 ± 0.55 grades on the Guillon Scale (*p* = 0.70). Examples of the interferometric lipid pattern differences are presented in Figure 2.

### 3.3. Aqueous Layer Quantity

**Figure 3:**
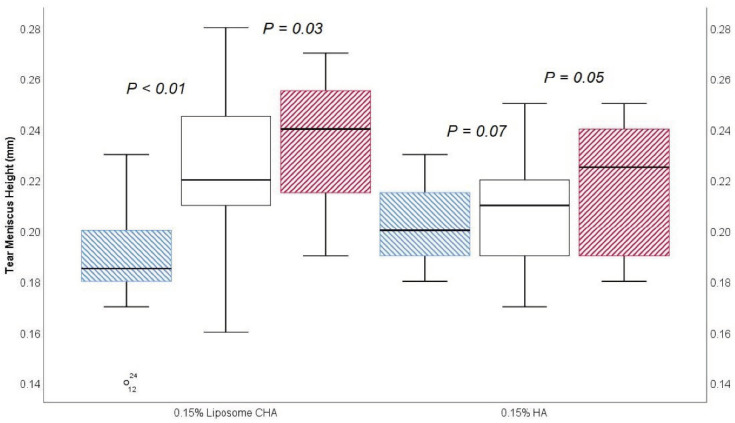
Box and plot of tear meniscus height assessment before (blue), 30 min (white), and 45 min (red) after eyedrop instillation.

Tear meniscus height assessment differences between the 0.15% LCHA group and the 0.15% standard HA group prior to eyedrop instillation and 30 min and 45 min after eyedrop instillation are presented in Table 2. In a longitudinal approach, for the 0.15% LCHA group, the change between the previous assessment and 30 min was an increase of 0.03 ± 0.01 mm (*p* < 0.01), and the change between the 30-min and 45-min assessments was an increase of 0.01 ± 0.02 mm (*p* = 0.03). Regarding the standard HA group, the change between the previous assessment and 30 min was 0.00 ± 0.01 mm (*p* = 0.07), and the change between the 30-min and 45-min assessments was 0.00 ± 0.01 mm (*p* = 0.05). A box and whisker plot of the tear meniscus height assessment is presented in Figure 3.

### 3.4. Mucin Layer Integrity

**Figure 4:**
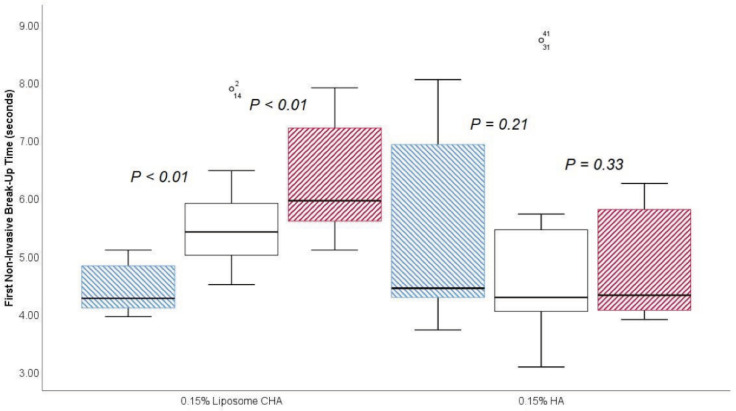
Box and plot of the first noninvasive break-up time between previous (blue), 30 min (white), and 45 min (red) after eyedrop instillation.

The noninvasive first and mean break-up time differences between the 0.15% LCHA group and the 0.15% standard HA group prior to eyedrop instillation and 30 min and 45 min after eyedrop instillation are presented in Table 2. In a longitudinal approach, for the first NIBUT in the 0.15% LCHA group, the change between the previous assessment and 30 min was an increase of 1.14 ± 0.90 s (*p* < 0.01), and the change between the 30-min and 45-min assessments was an increase of 0.71 ± 0.57 s (*p* < 0.01). Regarding the standard HA group, the change between the previous assessment and 30 min was a decrease of 0.50 ± 1.61 s (*p* = 0.21), and the change between the 30-min and 45-min assessment was 0.00 ± 1.33 s (*p* = 0.33). A box and whisker plot of the first NIBUT assessment is presented in Figure 4.

**Figure 5:**
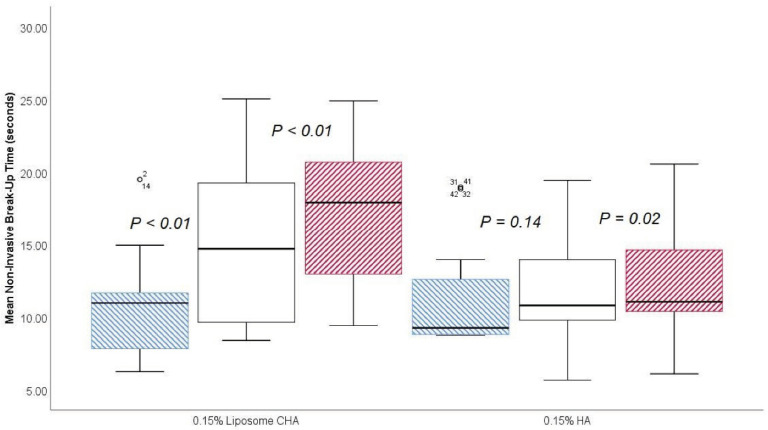
Box and plot of mean noninvasive break-up between previous (blue), 30 min (white), and 45 min (red) after eyedrop instillation.

The mean NIBUT for the 0.15% LCHA group for the change between the previous assessment and 30 min was an increase of 4.38 ± 2.89 s (*p* < 0.01), and the change between the 30-min and 45-min assessments was an increase of 1.99 ± 3.03 s (*p* < 0.01). For the standard HA group, the change between the previous assessment and 30 min was an increase of 0.56 ± 1.85 s (*p* = 0.14), and the change between the 30-min and 45-min assessment was an increase of 0.51 ± 1.18 s (*p* = 0.02). A box and whisker plot of the mean NIBUT assessment is presented in Figure 5.

## 4. Discussion

### 4.1. Limbal and Bulbar Redness

In our study, non-invasive diagnosis of the ocular surface was made. The OSA allows a quick and detailed analysis of the composition of the tear; it also identifies the type of dry eye disease and determines which layers should be treated according to the type of deficiency. The use of noninvasive diagnostic instruments is recommended for the evaluation of the tear film by improving the repeatability of the measurement [5,8]. Some of the works reviewed studied the effects of hyaluronic acid combined with other molecules in subjects with dry eye [1,2,3,24,25,26]. To our knowledge, our study is the first to show the benefits of the combination of cross-linked hyaluronic acid, liposomes, and crocin on the anterior surface of the eye in dry eye disease-symptomatic subjects. Our results show increased lipid layer thickness and tear break-up time after the instillation of 0.15% crosslinked hyaluronic acid with liposomes and crocin. LBRC is a characteristic clinical sign of DED that is associated with vasodilation of the conjunctival microvasculature. Evaluation of this conjunctival clinical sign is important in assessing the progression or improvement of DED [27].

In a longitudinal approach, our results showed a decrease over time in bulbar redness scores in both the LCHA and HA groups. This decrease was only statistically significant between 30 min and 45 min (0.16 ± 0.38 grades on the Efron Scale) (*p* = 0.04) in the LCHA group. Molina-Solana et al. [25] showed the same trend in their investigation, in which they described a statistically significant decrease after one month of treatment with hyaluronic acid 0.4% and galactoxyloglucan 0.2% in a group of dry eye patients. HA has an antioxidant cytoprotective effect on corneal epithelial cells. It is a molecule that improves the function of the corneal epithelial barrier and has beneficial effects on the regeneration of the corneal epithelium in the long term [28]. LCHA has higher viscosity and mucoadhesive properties and, in the long term, increases hydration and reduces inflammation related to friction [16]; moreover, prolonged treatment would be able to significantly reduce bulbar redness.

### 4.2. Lipid Layer Thickness

LLT is produced mainly in the Meibomian glands and contains mostly (80–90%) low polarity lipids (wax esters, cholesterol, and triglycerides) that are located in the outermost part, and 10–20% high polarity lipids (free fatty acids, glycolipids, lecithins, and phospholipids) that are located in the deepest part, orienting their hydrophilic polar group toward the aqueous phase [29]. The main function of the lipid phase is to prevent the evaporation of the aqueous phase [30]. Alterations of the lipid layer can be both qualitative and quantitative and are the cause of many of the changes related to dry eye [21,31]; therefore, maintaining the stability of the lipid layer is considered essential in clinical practice to prevent evaporation and improve the symptoms of these patients. Currently, it is common to include lipids in the formulation of eye drops [21] to restore the altered lipid layer, reduce the evaporation of the tear film, and relieve the symptoms of DED.

The instillation of a single lipid-containing artificial tear drop is capable of increasing the thickness of the lipid layer [32,33]. Our results confirmed this trend and showed a statistically significant increase in the LCHA group at 30 and 45 min after drop instillation compared to the HA group. In a longitudinal approach, this trend was also maintained over time, showing statistically significant increases in LLT at 30 min (*p* < 0.01) and 45 min (*p* = 0.04) in the LCHA group.

LCHA eye drops contain liposomes, which are spherical structures that form spontaneously when lipids are dispersed in an aqueous medium. Phospholipids are commonly used in the manufacture of liposomes [9] to increase the thickness of the lipid layer. Our results are consistent with studies that show an increase in LLT after the instillation of drops that include lipids in their composition [32,34,35,36]. However, in the HA group, the thickness of the lipid layer decreased at 30 min (0.50 ± 0.51 degrees on the Guillon Scale) (*p* = 0.01) and 45 min (0.04 ± 0.55 degrees on the Guillon Scale) (*p* = 0.70). HA is an excellent ocular lubricant due to its high capacity to retain water [16,37]. By instilling HA eye drops, the volume of the aqueous layer increases, which improves the distribution of the lipid layer [38], producing the LLT thinning shown by our results. Goto et al. [38] reported that dry eye disease patients present a thicker lipid layer due the lack of aqueous tear film. Consequently, HA instillation replenishes the aqueous part of the tear and therefore the lipid layer returns to the original volume according to our reported results. In their research, Li et al. [39] reported a decrease in LLT in a group of patients without alteration of the lipid layer after the administration of HA. In addition, they showed that the lower the concentration of HA, the higher the LLT increases in subjects with lipid deficiencies.

### 4.3. Tear Meniscus Height

TMH represents a high percentage of the volume of the tear film on the ocular surface. A decrease in TMH is related to dry eye syndrome due to a lack of aqueous secretion; therefore, its measurement is essential in the diagnosis of DED [40]. Our results showed an increase in TMH in both groups, which was slightly higher in the LCHA group. Other authors have come to similar results, all of which indicate an increase in TMH after the use of HA [41,42,43]. The slight difference in the LCHA group may be due to its higher viscosity and therefore longer contact time on the ocular surface that LCHA provides.

### 4.4. First and Mean Noninvasive Break-Up Time

The mucin layer is the highly hydrated inner layer of the tear film that covers the corneal and conjunctival epithelium. It is comprised of hydrated glycoproteins secreted by the goblet cells of the conjunctiva. Mucin contributes to water retention on the ocular surface [44]. Our study included two tear break-up times using the OSA, the first break-up time (FNIBUT) and the mean break-up time (MNIBUT), which is the mean of all tear film break-ups that occur over the entire cornea. There was an increase in FNIBUT in the LCHA group between instillation and the following 30 and 45 min with statistically significant values (*p* < 0.01). However, in the HA group, FNIBUT decreased 30 min after eye drop instillation. Regarding MNIBUT, the LCHA group also showed increases with statistical significance at 30 and 45 min after instillation, while, in the HA group, the average time increased over time, but only with statistical significance at 45 min after instillation.

To our knowledge, the active ingredients that promote mucin secretion are diquafosol sodium and rebamipide, which were recently introduced in the Japanese market [45]. HA is ineffective when the cause of DED is due to alteration of the mucin layer, since it does not act on goblet cells, but its high capacity to retain water provides beneficial effects that improve dry eye symptoms. This capacity is increased when combined with other molecules (galactoxyloglucan, cyanocobalamin, coenzyme Q10, vitamin E, crocin, liposomes, etc.) [1,2,3,16,24,46] or when formulated as LCHA. The results of our study show how both FNIBUT and MNIBUT were improved in the LCHA group compared to the HA group. A similar trend has been confirmed by numerous investigations [1,2,3,16,24,25,46] showing that the use of HA and LCHA drops in combination with other agents improves dry eye-related symptomatology, including tear break-up time values.

### 4.5. Strengths and Limitations

Regarding the strengths, this is the first study to report tear film stability parameters within a liposome and crosslinked hyaluronic acid eye drop. In addition, noninvasive and objective measurements were included. Considering the limitations, although both groups were not significantly different at baseline, some clinical differences were achieved between the LCHA and HA groups. Longer follow-up times and larger sample sizes should confirm these results. In addition, double-blind design research should reduce the methodology bias.

## 5. Conclusions

Crosslinking hyaluronic acid with liposomes and crocin significantly increases the permanence and stability of lipid, aqueous, and mucin tear film layers. In a short-term period, liposome and crosslinked hyaluronic acid achieved better first and mean noninvasive break-up times than standard hyaluronic acid. Liposomes enhance the interferometry lipid thickness, while standard hyaluronic acid dilutes the lipid content.

## Figures and Tables

**Table 1:** Demographics and baseline subjects’ characteristics.

Variable	0.15% Liposome-CHA (*n* = 30)	0.15% HA (*n* = 30)	*p* Value
Male (%) Female (%)	6 (25.0) 18 (75.0)	12 (50.0) 12 (50.0)	0.08
Age (years)	20.67 ± 2.18 (18.00 to 24.00)	21.42 ± 0.77 (20.00 to 22.00)	0.14
Sphere Refraction (D)	−0.95 ± 1.66 (−3.50 to +0.50)	0.14 ± 1.41 (−2.00 to 3.25)	0.29
Cylinder Refraction (D)	−0.37 ± 0.38 (−1.25 to 0.00)	−0.45 ± 0.54 (−1.75 to 0.75)	0.35
Axis Refraction (Degrees)	76.17 ± 81.16 (0.00 to 180.00)	94.00 ± 58.92 (5.00 to 175.00)	0.20
CDVA (Log MAR)	−0.04 ± 0.07 (−0.10 to +0.10)	−0.03 ± 0.08 (−0.10 to +0.10)	0.90
CDVA (Snellen)	20/18.50 ± 3.42 (20/16 to 20/25)	20/18.71 ± 3.66 (20/16 to 20/25)	0.90
Schirmer (mm)	9.83 ± 8.89 (0.00 to 30.00)	15.46 ± 9.15 (0.00 to 35.00)	0.11
BUT (seconds)	6.92 ± 2.71 (3.00 to 10.00)	8.21 ± 3.90 (3.00 to 13.00)	0.24
OSDI (score)	18.57 ± 10.96 (6.81 to 41.66)	21.26 ± 17.45 (5.40 to 47.92)	0.96
SPEED (score)	7.50 ± 5.09 (2.00 to 17.00)	10.42 ± 6.15 (2.00 to 19.00)	0.11

CHA: Crosslinked Hyaluronic Acid, HA: Hyaluronic Acid, D: Diopter, CDVA: Corrected Distance Visual Acuity, OSDI: Ocular Surface Disease Index, SPEED: Standard Patient Evaluation of Eye Dryness.

**Table 2:** Ocular surface analyzer comparison previous and after both eyedrop instillation.

Variable		0.15% Liposome-CHA (*n* = 30)	0.15% HA (*n* = 30)	*p* Value
Previous to eyedrop	Conjunctival Redness (Efron Scale)	0.67 ± 0.76 (0.00 to 2.00)	0.83 ± 0.56 (0.00 to 2.00)	0.27
	Lipid Layer Thickness (Guillon Pattern)	1.00 ± 0.83 (0.00 to 2.00)	1.62 ± 0.71 (1.00 to 3.00)	0.01
	Tear Meniscus Height (Millimeters)	0.18 ± 0.02 (0.14 to 0.23)	0.20 ± 0.15 (0.18 to 0.23)	0.01
	First NIBUT (seconds)	4.44 ± 0.40 (3.95 to 5.10)	5.30 ± 1.42 (3.72 to 8.04)	0.09
	Mean NIBUT (seconds)	10.85 ± 3.62 (6.25 to 19.50)	11.33 ± 3.77 (8.76 to 18.89)	0.96
30 min after	Conjunctival Redness (Efron Scale)	0.67 ± 0.48 (0.00 to 1.00)	0.75 ± 0.44 (0.00 to 1.00)	0.53
	Lipid Layer Thickness (Guillon Pattern)	1.83 ± 0.70 (0.00 to 3.00)	1.13 ± 0.61 (0.00 to 2.00)	<0.01
	Tear Meniscus Height (Millimeters)	0.22 ± 0.03 (0.16 to 0.28)	0.21 ± 0.02 (0.17 to 0.25)	0.03
	First NIBUT (seconds)	5.59 ± 0.89 (4.50 to 7.88)	4.77 ± 1.45 (3.08 to 8.72)	<0.01
	Mean NIBUT (seconds)	15.24 ± 5.40 (8.40 to 25.04)	11.89 ± 3.64 (5.68 to 19.44)	0.12
45 min after	Conjunctival Redness (Efron Scale)	0.50 ± 0.51 (0.00 to 1.00)	0.75 ± 0.44 (0.00 to 1.00)	0.07
	Lipid Layer Thickness (Guillon Pattern)	2.00 ± 0.83 (1.00 to 3.00)	1.17 ± 0.63 (0.00 to 2.00)	<0.01
	Tear Meniscus Height (Millimeters)	0.23 ± 0.02 (0.19 to 0.27)	0.21 ± 0.02 (0.18 to 0.25)	0.03
	First NIBUT (seconds)	6.30 ± 0.94 (5.10 to 7.90)	4.77 ± 0.89 (3.90 to 6.25)	<0.01
	Mean NIBUT (seconds)	17.23 ± 5.11 (9.41 to 24.90)	12.41 ± 4.18 (6.11 to 20.56)	<0.01

CHA: Crosslinked Hyaluronic Acid, HA: Hyaluronic Acid, NIBUT: Non-Invasive Break-Up Time.

## Data Availability

The data presented in this study are available on request from the corresponding author. The data are not publicly available due to their containing information that could compromise the privacy of research participants.
